# Pathogenic Variability of *Colletotrichum* Isolates From the Southern Areas of Coffee (*Coffea arabica* L.) Producing in Ethiopia

**DOI:** 10.1155/ijfo/5594372

**Published:** 2025-08-29

**Authors:** Ano Wariyo Negasso

**Affiliations:** ^1^ Plant Protection Department WondoGenet Agricultural Research Center, Ethiopian Institute of Agricultural Research (EIAR), Addis Ababa, Ethiopia, eiar.gov.et

**Keywords:** characteristics, conidia, green berry, pathogens, virulence

## Abstract

Arabica coffee plays a crucial role in the Ethiopian economy. Unfortunately, the production of this important crop is severely affected by coffee berry disease (CBD), which is caused by the fungus *Colletotrichum kahawae* Waller and Bridge. Information regarding the pathogenic variability of *Colletotrichum* isolates in southern Ethiopia is limited. Consequently, this study is aimed at characterizing and assessing the pathogenicity of representative *Colletotrichum* isolates from various coffee‐producing regions in southern Ethiopia. Eighteen representative isolates of *Colletotrichum* from the study area, along with one isolate from Gera, were obtained from infected green coffee berries. These isolates exhibited significant variations in their cultural and morphological characteristics, as well as differences in incubation periods and pathogenicity. The findings from the cultural and morphological analysis indicated variability in colony color and type, mycelial growth rates, and conidial production and size. The growth rate of mycelia varied significantly (*p* < 0.001), with measurements ranging from 2.05 to 4.14 mm per 24 h. Additionally, conidial size exhibited notable differences (*p* < 0.001), with widths varying from 2.31 to 4.41 *μ*m and lengths spanning from 10.15 to 12.17 *μ*m. Conidial production also showed considerable variation (*p* < 0.001), ranging from 157.21 to 418.12 conidia/mL. Among the detected 18 isolates, 13 were pathogenic to the susceptible coffee variety 370, displaying differences in disease reaction percentages that ranged from 75.00% to 100.00%. Furthermore, the incubation period showed substantial variation among isolates, ranging from 5.0 to 7.00 days. In conclusion, the aggressive isolate FG should be utilized for screening purposes when assessing coffee germplasms for resistance to CBD in southern Ethiopia.

## 1. Introduction

Coffee is one of the most vital commodities in the global agricultural commerce and a major source of revenue for many coffee‐producing nations [1, 2]. It is currently grown on more than 10.6 million hectares of land in the world’s subtropical regions in more than 80 countries. With a gross domestic product of 4%–5%, total government revenue of 10%, total agricultural production of 10%, total exports of 40%, and total export earnings of 25%–30%, coffee is an essential crop for Ethiopia’s economy [3]. Ethiopia is Africa’s top producer and the world’s fifth‐largest producer of Arabica coffee [4].

However, coffee berry disease (CBD) significantly reduces Ethiopia’s crop’s economic output. In regions with high rainfall, high humidity, and high altitude, as well as when sensitive cultivars are utilized, yield losses from this disease can vary from 24% to 30%, but they can potentially approach 100% [5, 6]. The very aggressive and specialized CBD is caused by a hemibiotrophic anamorphic fungal pathogen known as *Colletotrichum kahawae* Waller and Bridge (synonym: *Colletotrichum coffeanum* Noack; teleomorph: *Colletotrichum cingulata*) [2, 6]. In the major *C. arabica*–growing nations, such as those in Asia and America, it is classified as a quarantine pathogen [7]. The disease is a common anthracnose that affects all stages of coffee berries, from flower to ripe fruit, with the greatest losses occurring between green pinhead berries and red cherries, along with occasional impacts on leaves and branch bark [1].

Numerous research studies have been conducted in Ethiopia examining the diversity of *Colletotrichum* species associated with coffee berries, focusing on cultural, morphological, and pathogenic characteristics [1, 5, 8–12]. These studies have highlighted unique traits that differentiate *C. kahawae* subsp. *kahawae* from other *Colletotrichum* species, including its relatively slow growth rate and its capacity to specifically infect green coffee berries, leading to CBD. Furthermore, variations in pathogenicity among *C. kahawae* isolates may arise from differences in cultural appearance, growth rates, and morphological traits (such as sporulation capacity, conidial size, and shape) across various media, as well as their overall aggressiveness [5, 9–12].

In addition to the introduction of 42 resistant coffee varieties over the years, efforts have been directed toward enhancing the genetic foundation of resistance. However, these initiatives have been challenged by the frequent variations of pathogens—particularly the Angefa cultivar found in southern Ethiopia [13]. A comprehensive understanding of the genetic diversity within the pathogen responsible for CBD could facilitate the development of coffee varieties with adequate disease resistance [14]. The severity of CBD in Ethiopia varies by region due to factors like susceptible coffee types, environmental conditions, and the virulence of *C. kahawae* isolates [15]. Derso and Waller [5] proposed that evaluating the profiles of various isolates from different coffee types across the country over time could help assess the pathogenic variability of isolates from different regions. Research on characterization and pathogenicity of *Colletotrichum* species in southern Ethiopia has been scarce, prompting this study to characterize and evaluate the pathogenicity of isolates from diverse coffee‐producing areas in the region. Therefore, this study is aimed at characterizing and evaluating the pathogenicity of selected *Colletotrichum* isolates gathered from different coffee‐growing areas in southern Ethiopia.

## 2. Materials and Methods

### 2.1. Description of the Study Sites

The agroecologic descriptions of the survey districts from where samples were collected are depicted hereunder (Table 1). The laboratory research was carried out in a laboratory and growth room at the Jimma Agricultural Research Center (JARC) during the 2021 cropping season.

**Table 1:** Geographic descriptions of the six study districts in southern Ethiopia during the long rainy season July to August 2021.

**Region/zone**	**District**	**Altitude (m.a.s.l.)**	**Lat. (N)**	**Long. (E)**	**Annual rain fall (mm)**	**Relative humidity (%)**	**Mean temperature (°C)**		
							**Min**	**Max**	**Mean**
Sidama	Dale	1708–1904	6.2	38.5	1350.2	73.3	6.6	29.5	18.0
Sidama	Shebedino	1725–1909	6.6	38.4	1181.2	67.0	10.0	35.1	22.5
Sidama	Aletawondo	1853–1961	6.9	38.5	1372.3	73.0	4.4	29.2	16.8
South Ethiopia (Gedeo zone)	Wonsho	1820–2002	6.2	38.3	1224.0	69.9	8.2	32.9	20.5
South Ethiopia (Gedeo zone)	Wonago	1745–1980	6.7	38.4	1400.4	76.0	5.2	27.9	16.6
South Ethiopia (Gedeo zone)	Yirgacheffe	1809–2071	6.7	38.5	1478.3	75.9	5.3	27.9	16.6

Abbreviation: m.a.s.l., meters above sea level.

### 2.2. Sample Collection

**Figure 1:**
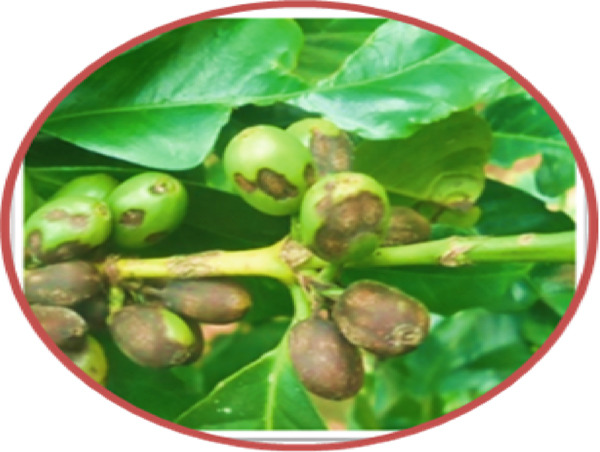
Typical symptoms of coffee berry disease on green coffee berries samples collected for laboratory analyses from farmers’ fields during the 2021 cropping season in southern Ethiopia.

During the July–August 2021 cropping season [8], a collection was made of infected green coffee berry specimens (15–20) exhibiting active black sunken lesions (Figure 1). These specimens were gathered from two regions/zones across six districts, with three farms selected per district, located in the southern coffee‐producing regions of Ethiopia (Table 1). Using sterilized forceps, samples were obtained and placed into perforated, sterile plastic bags. This process involved a total of 67 coffee farms, all of which were experiencing CBD (Figure 1). The samples were then delivered to the Plant Pathology Laboratory at JARC, where they were stored at 4°C prior to the isolation process.

### 2.3. Isolation and Identification of the Colletotrichum Isolates

The isolation and identification of the *Colletotrichum* pathogen isolates were conducted using infected green coffee berries, following the methodology outlined by Photita et al. [16]. In summary, 5 × 5 mm sections of the outer tissue from the margins of actively infected green berries (comprised of both healthy and infected portions) were excised and subjected to surface sterilization by soaking in a 5% sodium hypochlorite solution (NaOCl 0.05% W/V) for 1 min. Subsequently, the samples were rinsed three times in sterilized distilled water (SDW), with each rinse lasting 1 min. After allowing excess moisture to evaporate under a laminar flow hood for 1 h, five tissue fragments were placed onto prepared Petri dishes containing potato dextrose agar (PDA) supplemented with streptomycin (50 *μ*g mL^−1^). The Petri dishes were then sealed with Parafilm and incubated at 25°C for a duration of 7 to 10 days. To achieve further purification and identification, mycelial growth from the peripheral hyphal extensions was collected and transferred under sterile conditions to new Petri dishes containing PDA, a procedure that was repeated multiple times to obtain a pure culture. Following cultural and microscopic identification, referencing the identification keys and species descriptions from Barnett and Hunter [17] and Waller et al. [18], 18 representative pure cultures of *Colletotrichum* isolates and well‐known reference isolate from Gera (GR) hot spot areas (Gera, Oromia region, Ethiopia) of CBD [17] were successfully preserved using the 50% PDA slant method at 4°C for future studies. On some culture of fragment samples, some pathogens, like *Fusarium* spp., *Penicillium* spp., *Phoma* spp., and *Aspergillus* spp., were grown and then removed, and the Petri dish was sterilized to avoid contamination.

### 2.4. Macroscopic (Cultural) and Microscopic (Morphological) Characterization of the *Colletotrichum* Isolates

The pathogen isolates were cultivated on agar plates and subsequently analyzed for their cultural, morphological, and pathogenic characteristics following established protocols [19].

#### 2.4.1. Macroscopic (Cultural) Features

The cultural variations among 19 representative *Colletotrichum* isolates, including the Gera isolate, were analyzed by culturing on PDA with 0.04% streptomycin. The cultures were incubated at 25°C, with three replications arranged in a completely randomized design (CRD) to assess all the traits. Each isolate was evaluated for characteristics including mycelial radial growth, colony color, colony shape, and aerial mycelial growth. The mycelial (colony) radial growth (measured in millimeter) of each isolate was recorded daily from the reverse side of the Petri dishes over a 10‐day period, beginning on the 3rd day of incubation. The colony color on the upper side and the types of pigments on both the front and reverse sides of the Petri dish were assessed every 3 days using an RGB color chart. Consequently, the cultures were monitored for a total of 12 days. The vigor of the aerial mycelium growth of each isolate was observed on the upper surface of the plate after 10 days of culture on PDA and categorized as dense (regular), irregular (scarce), or very scarce. Additionally, the colony shape of each isolate was evaluated from the reverse side of the plate after 8 days of culture on PDA and classified into forms such as round, irregular, filamentous, rhizoid, or curled.

#### 2.4.2. Microscopic (Morphological) Features

Isolates were cultured on PDA medium in triplicate for a duration of 10 days, after which the conidial dimensions (length and width) were measured for 30 randomly selected conidia from each replicate per isolate. The length and width of the conidia were assessed using an ocular micrometer (micrometer) at a magnification of 40× with a compound microscope (Omax 40X–2500X, Asian). To determine the sporulation capacity of each isolate, cultures that were 10 days old on PDA were subjected to a washing procedure involving the addition of 10 mL of SDW. A sterilized scalpel was used to rub the cultures, which were then transferred to a 50‐mL sterilized beaker. The mixture was stirred thoroughly for 15 min using a magnetic stirrer to dislodge the spores from the entangled mycelium. Subsequently, the mycelium was filtered into another sterilized beaker using double‐layered cheesecloth. The concentration of conidia per milliliter was quantified using a hemocytometer.

### 2.5. Pathogenicity Test and Incubation Periods of the *Colletotrichum* Isolates

#### 2.5.1. Detached Berry Test (DBT)

Eighteen representative *Colletotrichum* isolates (mentioned under isolation and identification session) were assessed for their pathogenicity on detached green berries of the susceptible variety 370, following the methods outlined by Van der Vossen et al. [20]. Coffee berries, aged 15 weeks (expanding stage) from the date of flowering, were randomly collected from the bottom, middle, and top of the coffee tree [21]. The berries underwent surface sterilization using a 0.05% (W/V) NaOCl solution for 2 min, followed by three rinses with sterile distilled water, each lasting 2 min, and were then dried with a sterile cotton cloth. To prevent contamination from saprophytic fungi, the wounded stalk ends of the berries were excised using a sterile scalpel. For inoculation purposes, 12 berries per isolate were arranged in three rows within a plastic box lined with sterilized tissue paper, adhering to a CRD with three replications for each isolate.

##### 2.5.1.1. Inoculum and Inoculation Preparation Procedures

The mycelial cultures of each isolate, 10 days old, were washed by flooding with 10 mL of SDW. Following this, the cultures were gently scraped with a sterilized scalpel and transferred to a 50‐mL sterilized beaker for conidia harvesting. The resulting suspension from each isolate was agitated using a magnetic stirrer for 15 min and then filtered through double layers of cheesecloth. This process was repeated, and the spore concentration of each suspension was adjusted to 2 × 10^6^ conidia/mL using hemocytometer [22, 23]. A volume of 20 *μ*L of the conidia suspension was then applied to the berries using a pipette, with intermittent shaking during inoculum placement. For control purposes, 20 *μ*L of SDW was applied to a separate set of berries. All the boxes used in the experiment were sealed to maintain the saturated humidity conditions required for disease development. The boxes were opened every 3 days for 10 min to allow for proper aeration of the berries. Data on infection were collected every 3 days, starting from the 3rd day postinoculation, when symptoms of CBD first became visible. After 14 days, each isolate’s reaction was noted as positive (+) for lesions or negative (−) for no reaction.

#### 2.5.2. Seedling Hypocotyl Laboratory Inoculation Tests

##### 2.5.2.1. Raising Coffee Seedlings

Coffee seedlings were raised in growth room from freshly picked seeds of susceptible variety 370. Ripened cherries were picked from mother trees in the field and dried under shade after removing the pulp by hand. After removing the parchment, the seeds were soaked in SDW and kept for 48 h. Thereafter, seeds were sown (30–40 seeds per box) in heat sterilized and moistened sandy soil in disinfected plastic boxes each with 2295 cm^3^ capacity arranged on benches and covered with chip wood in growth room.

##### 2.5.2.2. Inoculation of Coffee Hypocotyls in the Laboratory

Two days before inoculating the hypocotyls at unfolding stage (i.e., 6 weeks after sowing), the temperature was adjusted to 20°C, and seedlings were sprayed with SDW and covered with plastic sheet for 48 h to obtain 100% relative humidity. Then, the coffee hypocotyls were inoculated with conidial suspension by stem brushing procedure with fine camel hairbrush as described by Van der Graaff [15]. The second reinoculation was conducted 48 h after the first inoculation and following the same procedures. All the treated hypocotyls were immediately covered with transplant plastic sheet to create humid condition for infection and maintained in cool place with a mean temperature of 20 ± 2^°^C in a growth room.

The reaction of each hypocotyl was assessed at 3, 5, 7, 10, 14, and 21 days after inoculation using the 0–4 disease scale symptom classifications and assessment key based on measured percentage of affected area employed by Van der Graaff [15], where 0 = no symptom, 1 = from very tiny to one or two narrow brown lesion up to 0.5 mm wide, 2 = more than two brown lesions or brown coalescing lesions exceeding 0.5 mm, black dots if present are rare, 3 = wide brown lesions with numerous black dots and/or black lesion may completely surrounded the stem but the top remain alive, and 4 = black lesion girdling the stem and top killed. A disease index reaction (DIR) for each accession was described as a percentage of the maximum possible infection using the following formula [15]:

DIR=w+234x+y+z×1004v+w+x+y+z

where *v* is the number of hypocotyls in Class 0, *w* is the number of hypocotyls in Class 1, *x* is the number of hypocotyls in Class 2, *y* is the number of hypocotyls in Class 3, and *z* is the number of hypocotyls in Class 4.

### 2.6. Statistical Analysis

The data were analyzed using ANOVA with the SAS Version 9.4 software package [24]. For mean comparison of treatment effects, Duncan’s multiple range test (DMRT) was employed at a significance level of 5%. Additionally, Pearson correlation analysis was conducted to assess the relationships among pathogen characteristics, and pathogenicity, utilizing the SAS software (PROC procedure).

## 3. Results

### 3.1. Macroscopic Features of the *Colletotrichum* Isolates

**Figure 2:**
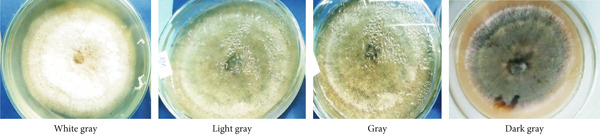
Description of the aerial mycelial (colony) colors from obverse side of the Petri plate.

In this study, we examined and identified the macroscopic characteristics of representative isolates of the *Colletotrichum* pathogen, including colony appearance, color, and the radial growth rate of mycelium (Table 2 and Figure 2). Visual assessments of the upper surface of the culture plates revealed that 31.6% of the isolates exhibited a scarce aerial mycelial growth, 47.4% demonstrated dense growth, and 21.1% were categorized as very scarce (Table 2). Additionally, the colony colors varied on both sides of the cultured plates. On the front side, we observed the following distribution of colors: 36.8% white gray, 5.3% light gray, 15.8% (including dark white gray, dark gray, and floral white), and 10.5% cottony white (Table 2 and Figure 2). Conversely, the back sides predominantly displayed Navajo white and light goldenrod colors, each accounting for 36.8%, followed by 15.8% with dark olive green. Overall, the majority of isolates exhibited a whitish mycelial color during the initial 4–6 days of incubation, transitioning to light gray colony colors after 1 week on PDA (Table 2).

**Table 2:** Macroscopic identification of *Colletotrichum* isolates.

**Isolate code**	**Colony types**	**Colony color**		**Colony growth (mm/day)**
		**Top**	**Reverse**	
HK01	Dense	Light gray	Light golden rod	3.10^cd^
AD	Dense	White gray	Navajo white	3.12^cd^
MG	Very scarce	Dark white gray	Light golden rod	2.18^f^
TR01	Very scarce	Dark white gray	Navajo white	2.21^f^
HM01	Dense	White gray	Pale golden rod	3.11^cd^
SK	Scarce	Dark gray	Dark olive green	3.27^cd^
TT01	Dense	Floral white	Navajo white	3.52^bc^
MF	Very scarce	Cottony white	Lemon chiffon	2.18^f^
MF02	Very scarce	Floral white	Navajo white	2.05^f^
BB01	Scarce	White gray	Navajo white	2.40^ef^
MK01	Dense	Cottony white	Lemon chiffon	3.90^ab^
MK02	Dense	Dark white gray	Light golden rod	3.78^ab^
WG	Dense	White gray	Dark olive green	3.06^cd^
BBK01	Scarce	Dark gray	Light gray	2.36^ef^
AW02	Scarce	White gray	Navajo white	2.26^f^
HB	Scarce	Floral white	Navajo white	2.77^de^
HB03	Scarce	White gray	Pale golden rod	2.96^d^
FG	Dense	White gray	Dark olive green	4.11^a^
GR	Dense	Dark gray	Light gray	4.14^a^
Mean	—	—	—	2.97
CV (%)	—	—	—	9.47

*Note:* Means followed with the same letters are not significantly different from each other (DMRT; at *p* < 0.05).

The results of the mycelial growth analysis indicated a significant (*p* < 0.001) difference among the isolates (Table 2). The mean radial mycelial growth rates varied between 2.05 and 4.14 mm per 24 h. The isolate derived from the Gera (GR) exhibited the highest growth rate at 4.14 mm/day, making it the fastest among all tested isolates, although it did not differ significantly from the FG isolate, which recorded a growth rate of 4.11 mm/day. Conversely, the isolate MF02 demonstrated the slowest mean radial growth rate at 2.05 mm/day, followed closely by the MF, MG, and TR01 isolates, which showed no significant differences among them (Table 2).

### 3.2. Microscopic Features of the *Colletotrichum* Isolates

A highly significant (*p* < 0.001) variation was observed among the isolates concerning their conidial size (both length and width) and sporulation capacity (Table 3). The isolates of *Colletotrichum* exhibited a range of conidial lengths from 10.15 to 10.71 *μ*m and widths from 2.31 to 4.41 *μ*m. The average measurements recorded for conidial length and width across the isolates were 11.47 and 5.82 *μ*m, respectively (Table 3). Notably, isolate MF02 demonstrated the largest mean conidial length at 12.71 *μ*m, while the smallest mean conidial lengths were observed in isolates FG and GR, measuring 10.15 and 10.72 *μ*m, respectively. In terms of conidial width, isolate MF exhibited the widest measurement of 4.41 *μ*m, whereas the narrowest widths were recorded in isolates GR and FG, measuring 2.31 and 2.44 *μ*m, respectively (Table 3). The sporulation capacity of selected *Colletotrichum* isolates was assessed using 10‐day‐old cultures, with conidial concentrations ranging from 157.21 to 418.12 × 10^4^ conidia/mL for isolates MF02 and FG, respectively (Table 3). Isolate FG produced a significantly higher amount of conidia (418.12 × 10^4^), followed closely by isolate GR, although the difference was not statistically significant. In contrast, the isolates MF02 (157.21 × 10^4^), MF (175.98 × 10^4^), and TR01 (177.70 × 10^4^) yielded the smallest quantities of conidia, which exhibited a highly significant difference when compared to the other isolates (Table 3).

**Table 3:** Microscopic identification of *Colletotrichum* isolates.

**Isolate code**	**Collection site**		**Sporulation capacity (no. of conidia/mL)**	**Conidial size (*μ*m)**	
	**District**	**Specific site (PA)**		**Length**	**Width**
HK01	Dale	Haleka	285.44^ef^	11.13^bcdef^	3.13^fg^
AD	Dale	Awada	240.96^gh^	11.90^abcde^	3.29^ef^
MG	Shebedino	Midregenet	179.94^ij^	12.17^abc^	3.78^cd^
TR01	Shebedino	Taramessa	177.70^j^	12.55^ab^	3.57^de^
HM01	Aletawondo	Homachew	319.66^d^	10.89^cdef^	2.90^gh^
SK	Aletawondo	Shiko	295.97^de^	11.10^bcdef^	3.24^ef^
TT01	Aletawondo	Titera	363.88^c^	10.71^def^	2.74^hi^
MF	Wonsho	Menafesha 01	175.98^j^	12.10^abcd^	4.41^a^
MF02	Wonsho	Menafesha 02	157.21^j^	12.71^a^	4.24^ab^
BB01	Wonsho	Bokaso	180.90^ji^	11.97^abcd^	3.89^cd^
MK01	Wonago	Mekanisa 01	400.07^ab^	10.25^f^	2.67^hi^
MK02	Wonago	Mekanisa 02	395.87^ab^	10.48^ef^	2.67^hi^
WG	Wonago	Kara Soditi	371.36^bc^	11.17^bcdef^	2.89^gh^
BBK01	Wonago	Bale Bukisa	258.62^fg^	11.82^abcde^	3.69^cd^
AW02	Yirgacheffe	Afursa Warebi	210.26^hi^	12.13^abcd^	4.01^bc^
HB	Yirgacheffe	Haro Batala 01	257.55^fg^	11.82^abcde^	3.66^cd^
HB03	Yirgacheffe	Haro Batala 02	235.07^gh^	12.14^abcd^	3.77^cd^
FG	Yirgacheffe	Fishagenet	418.12^a^	10.15^f^	2.44^ij^
GR	Gera	Gera	412.19^a^	10.72^cdef^	2.31^j^
Mean	—	—	280.88	11.47	3.33
CV (%)	—	—	6.34	6.45	5.82

*Note:* Means followed with the same letters are not significantly different from each other (DMRT; at *p* < 0.05).

### 3.3. Pathogenicity Test and Incubation Periods of the *Colletotrichum* Isolates

Out of the 18 *Colletotrichum* isolates examined, 13 were identified as *C. kahawae*, whereas four were identified as *Colletotrichum gloeosporioides*, and a single isolate was identified as *Colletotrichum acutatum* (Table 4). This finding suggests the presence of pathogenic variation across all isolates evaluated in both detached berry and hypocotyl tests. Specifically, the most severe disease reactions, quantified at 100.00%, were documented in the FG isolates originating from the Yirgacheffe and GR Gera locations.

**Table 4:** Pathogenicity and incubation periods among *Colletotrichum* isolates.

**Isolate code**	**Identified *Colletotrichum* species**	**Pathogenicity**			**Incubation period**
		**DBT reaction**	**Hypocotyl test**		
			**Reaction**	**DIR (%)**	
HK01	*C. kahawae*	+	+	90.63	6.00
AD	*C. kahawae*	+	+	84.69	7.00
MG	*C. gloeosporioides*	−	−	0.00	0.00
TR01	*C. gloeosporioides*	−	−	0.00	0.00
HM01	*C. kahawae*	+	+	93.91	6.00
SK	*C. kahawae*	+	+	90.07	6.00
TT01	*C. kahawae*	+	+	96.36	5.00
MF	*C. gloeosporioides*	−	−	0.00	0.00
MF02	*C. gloeosporioides*	−	−	0.00	0.00
BB01	*C. acutatum*	−	−	0.00	0.00
MK01	*C. kahawae*	+	+	98.10	5.00
MK02	*C. kahawae*	+	+	96.67	5.00
WG	*C. kahawae*	+	+	93.87	6.00
BBK01	*C. kahawae*	+	+	79.42	7.00
AW02	*C. kahawae*	+	+	75.00	7.00
HB	*C. kahawae*	+	+	87.17	6.00
HB03	*C. kahawae*	+	+	84.23	6.00
FG	*C. kahawae*	+	+	100.00	5.00
GR	*C. kahawae*	+	+	100.00	5.00
Control	—	−	−	0.00	0.00

*Note:* + and − stand for pathogenic and nonpathogenic of *Colletotrichum* spp. isolates to coffee green berry and seedling, respectively.

Abbreviations: DBT, detached berry test; DIR, disease index reaction.

^*^^*^= GR isolate.

In contrast, isolates MG, MF02, TR01, MF, and BB01 exhibited no disease response (Table 4). Green berries and hypocotyls, when inoculated with the pathogenic species (*C. kahawae*), manifested disease symptoms within 5–7 days postinoculation on detached berries and hypocotyls (Table 4, Figures 3 and 4). During the hypocotyl assays, small brown spots initially appeared, subsequently evolving into grayish‐brown lesions that coalesced into dark brown lesions. These lesions resulted in wilting and eventual death of the upper portion of the seedlings (Figure 4). The reisolation of the inoculated culture from the affected coffee berries and hypocotyls yielded the characteristic colony morphology of *C. kahawae*.

Figure 3Pathogenicity test of *Colletotrichum* isolates on detached berry test on susceptible coffee variety 370. (a) Isolates of *C. gloeosporioides* (MG). (b) Isolates of *C. acutatum* (BB01). (c) Control. (d–f) Isolates of *C. kahawae* (FG and GR), manifested disease symptoms on green berries.

**Figure:**
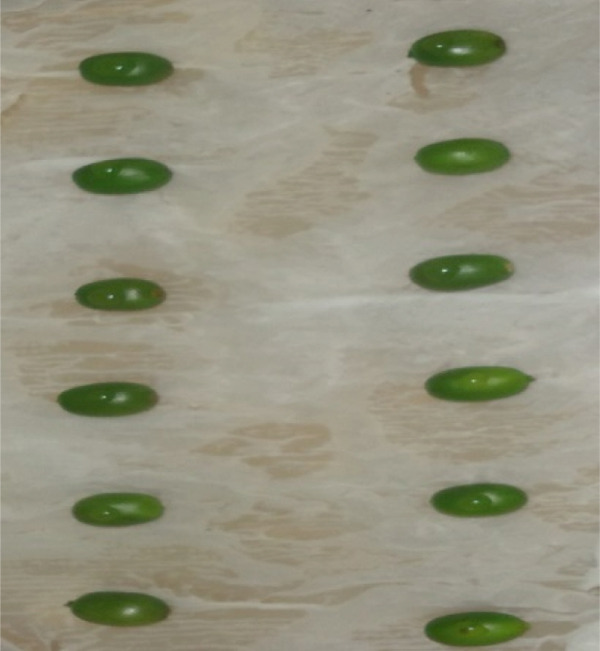
(a)

**Figure:**
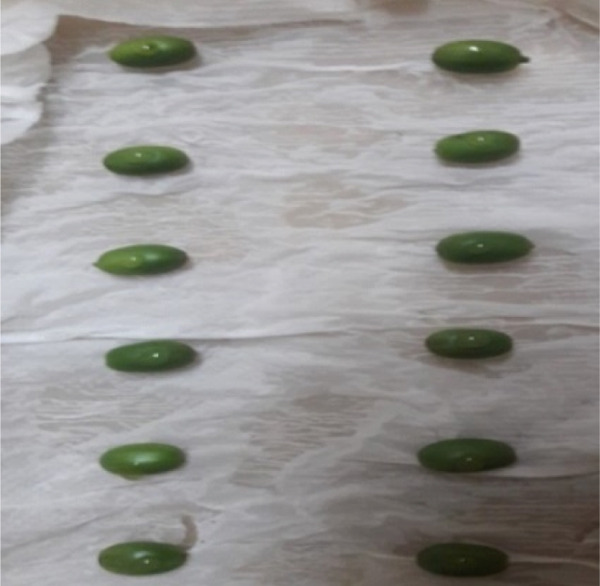
(b)

**Figure:**
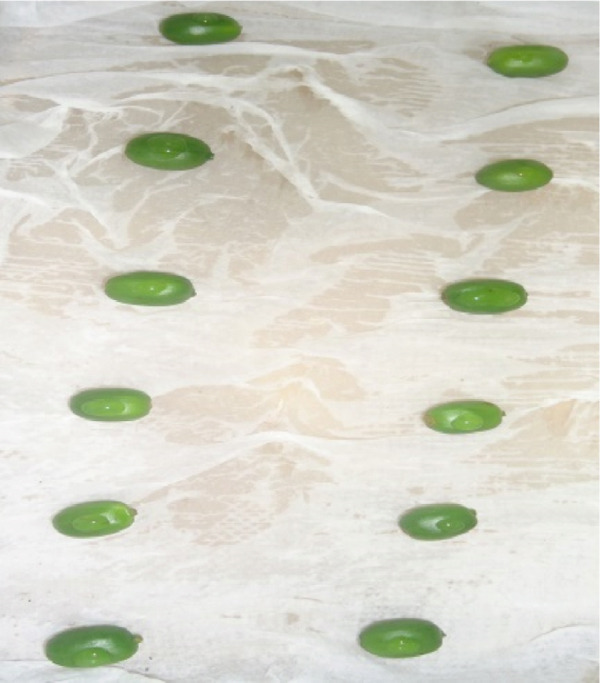
(c)

**Figure:**
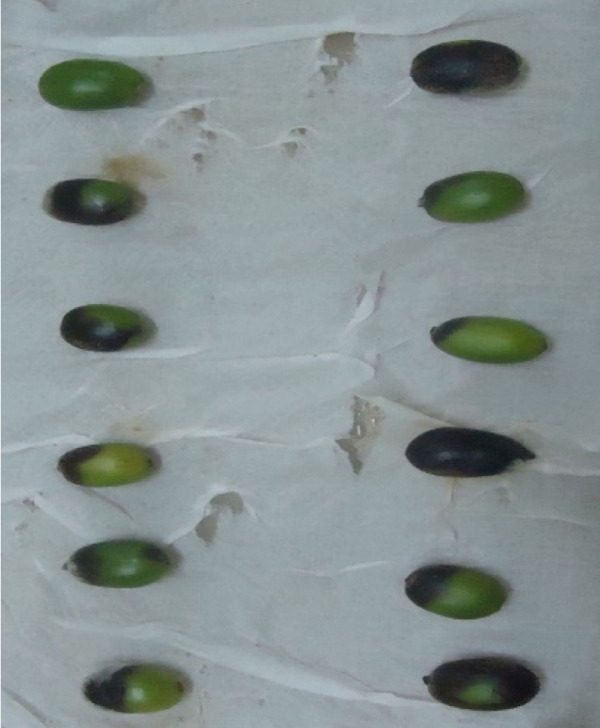
(d)

**Figure:**
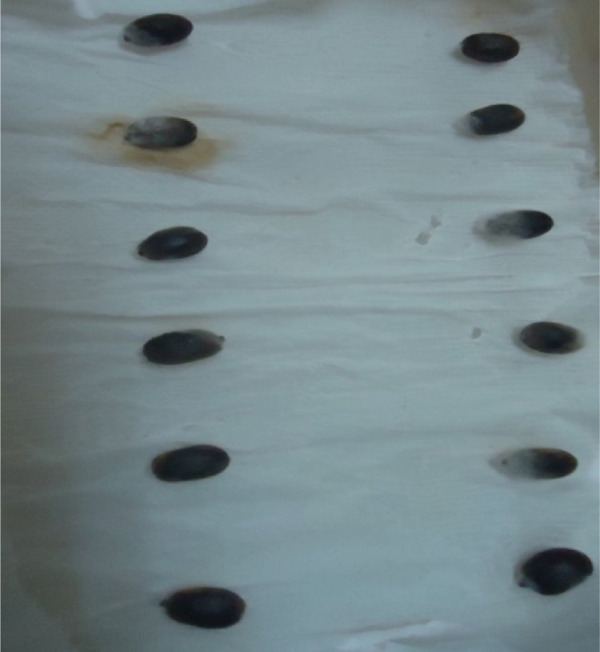
(e)

**Figure:**
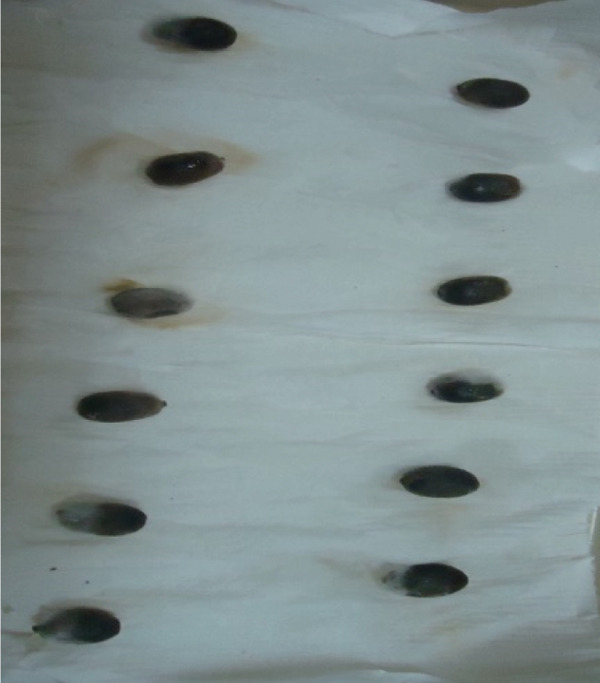
(f)

Figure 4Pathogenicity test of *Colletotrichum* isolates on seedling hypocotyls inoculation test on susceptible coffee variety 370. (a) Isolates of *C. gloeosporioides* (MG). (b) Isolates of *C. acutatum* (BB01). (c) Control. (d, e) Isolates of *C. kahawae* (FG and GR), manifested disease symptoms on seedlings.

**Figure:**
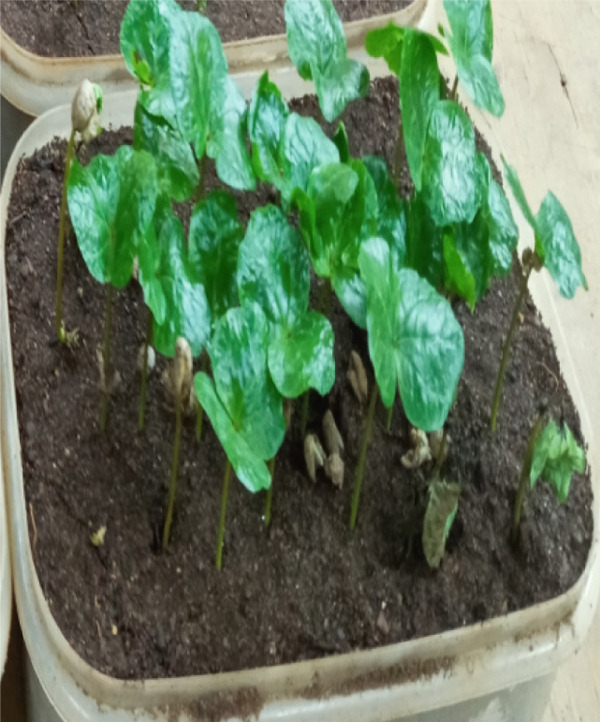
(a)

**Figure:**
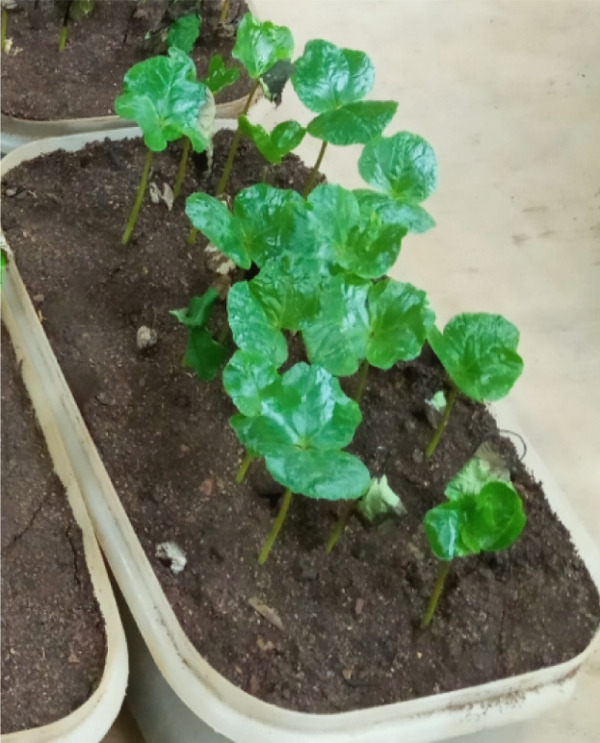
(b)

**Figure:**
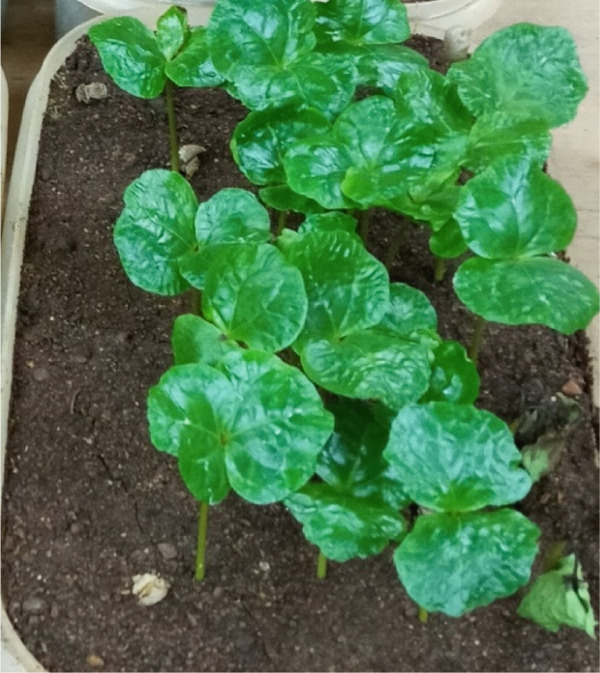
(c)

**Figure:**
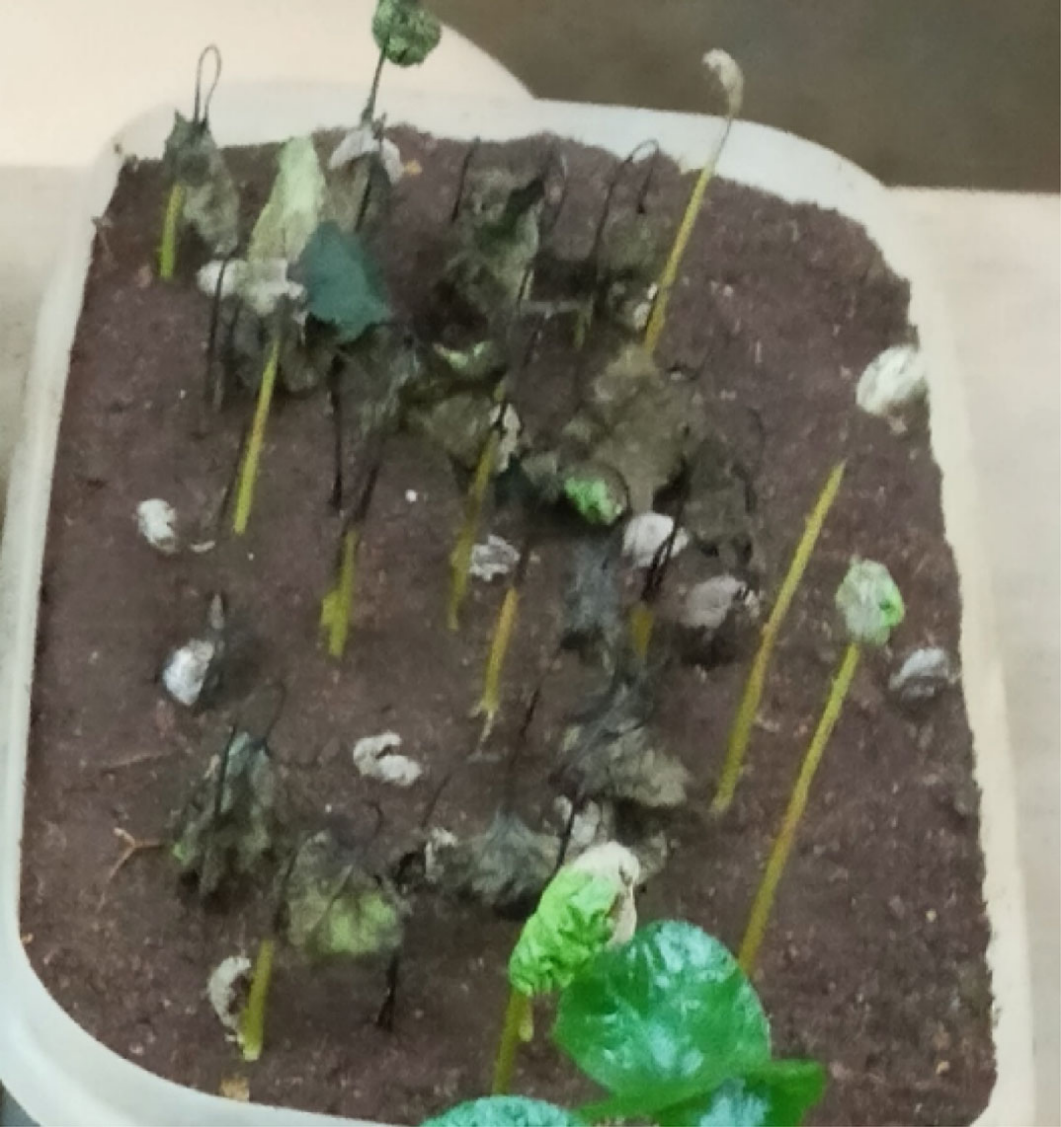
(d)

**Figure:**
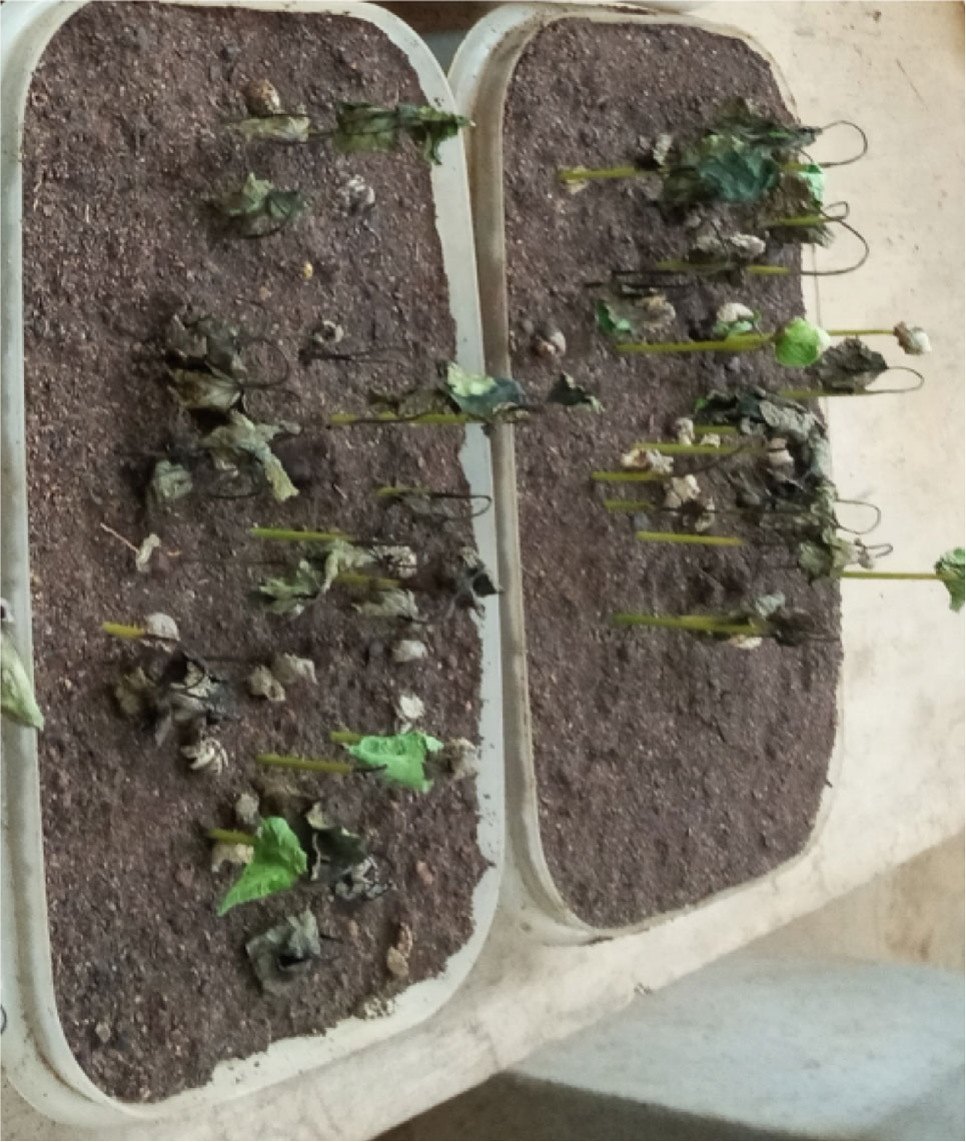
(e)

### 3.4. Association of Morphocultural Characteristics With the Pathogenicity Test of *C. kahawae* Isolates

The Pearson correlation analysis demonstrated a highly significant (*p* < 0.001) and strong positive correlation between the virulence of *C. kahawae* isolates and their colony (mycelial) growth rate (*r* = 0.92) as well as their sporulation capacity (*r* = 0.88), as summarized in Table 5. Conversely, the conidial size—specifically conidial width (*r* = −0.36) and length (*r* = −0.42)—exhibited an insignificant and no correlation with the virulence of the isolates (Table 5). These findings suggest that, unlike the size of fungal fruiting structures, rapid colony (mycelial) growth and increased sporulation capacity may enhance the aggressiveness of *C. kahawae* isolates in *C. arabica*.

**Table 5:** Pearson correlation analysis of morphocultural characteristics and pathogenicity of the *C. kahawae* isolates of southern Ethiopia.

	**Colony growth**	**Sporulation capacity**	**Width**	**Length**	**Pathogenicity**
Colony growth	1.00	0.88 ^∗∗^	−0.92 ^∗∗^	−0.87 ^∗∗^	0.92 ^∗∗^
Sporulation capacity		1.00	−0.95 ^∗∗^	−0.94 ^∗∗^	0.94 ^∗∗^
Width			1.00	0.92 ^∗∗^	−0.36 ^∗^
Length				1.00	−0.42^ns^
Pathogenicity					1.00

Abbreviation: ns, nonsignificant.

^*^Significant at <0.05 level.

^**^Highly significant at 0.01 level.

## 4. Discussion

### 4.1. Macroscopic (Cultural) and Microscopic (Morphological) Characteristics of *Colletotrichum* Isolates

Significant variations in cultural and morphological traits were observed among the *Colletotrichum* isolates obtained from prominent coffee‐growing regions in southern Ethiopia. Similarly, studies conducted by Biratu [8], Zeru et al. [25], Zenebe et al. [12], and Amare et al. [11] have reported the presence of cultural diversity among *Colletotrichum* isolates from coffee cultivation areas in Ethiopia. In agreement with these findings, Biratu and Hulluka [19] noted variations in the radial mycelial growth of *Colletotrichum* isolates, with average growth rates of 6.5 and 6.7 mm per 24 h on PDA. Notably, the current study yielded results that align closely with those of Zeru [26], Zenebe et al. [12], and Amare et al. [11]. Cultural characteristics serve as a valuable taxonomic tool for the identification of fungal species [27]. The *C. kahawae* isolates associated with coffee exhibit differences in mycelial growth rates, colony morphology, and growth patterns compared to other *Colletotrichum* isolates, which is pivotal for their classification based on visual observations from culture plates ([11], [28], and [12]). Kebati et al. [29] emphasized that conidial morphology has traditionally been prioritized over other taxonomic criteria in studies of the *Colletotrichum* genus. Given that *C. kahawae* is characterized by a slow mycelial growth rate on culture media in natural settings, this trait may serve as a distinguishing factor from other species and could indicate variability within the species itself ([11], [30], and [12]). Furthermore, *C. kahawae* displays consistent characteristics across its distribution range and is predominantly associated with coffee [6]. The pathogen’s unique infection dynamics and its ability to occupy ecological niches for development on green berries are also key distinguishing features that set it apart from other *Colletotrichum* species [31].

Moreover, all the isolates demonstrated variability in conidial length and width, consistent with findings from various researchers. For example, Biratu [8] reported a mean conidial length ranging from 13.5 to 19.3 *μ*m and a width of 2.9 to 5.2 *μ*m on PDA. Similarly, Kilambo [22] noted conidia lengths between 8 and 18 mm and widths from 2 to 6 mm, indicating an overlap in conidial sizes among the isolates, which complicates the differentiation of *C. kahawae* strains based solely on conidial dimensions [12]. The high conidia production observed is characteristic of the virulent *C. kahawae* isolates, providing a substantial inoculum source for disease progression. This observation aligns with the conclusions of Derso and Waller [5] and Zeru [26], who noted that conidial morphology serves as a key distinguishing feature of *C. kahawae* isolates compared to other *Colletotrichum* species and that sporulation potential may vary among isolates [11, 12]. Furthermore, Alemu et al. [9] found that isolates of *Colletotrichum* species yielded an average conidial count ranging from 2.4 × 10^5^ to 1.3 × 10^7^ on PDA.

### 4.2. Pathogenicity Test and Incubation Periods of *Colletotrichum* Isolates

Under laboratory conditions, the pathogenicity test and incubation period displayed considerable diversity across the examined *Colletotrichum* isolates. This disparity could be a result of the varying degrees of virulence or aggressiveness demonstrated by the isolates. Notably, isolate FG demonstrated the highest conidia production and exhibited more virulence compared to the other isolate. Consistently, Alemu et al. [9], Amare et al. [11], and Zenebe et al. [12] observed that certain *C. kahawae* strains displayed high virulence due to their enhanced sporulation capacity and the effective germination of conidia within host tissues. Derso and Waller [5] further emphasized that the DBT and hypocotyls test can be employed to assess the aggressiveness of various *C. kahawae* strains originating from the same or different geographic locations [12]. The virulence level, or aggressiveness of the pathogen, serves as a quantitative measure of disease progression over time, indicating that the most aggressive strain can reach a specific disease level more rapidly than its less aggressive counterparts. This progression can be quantified through metrics such as latent period, spore production, infection rates, lesion size, and overall disease severity [31].

### 4.3. Association of Morphocultural Characteristics With the Pathogenicity Test of *C. kahawae* Isolates

In this study, we observed significant variations in the association of morphological and cultural characteristics, with the results of pathogenicity tests on representative isolates of the pathogens. Particularly noteworthy were the isolates FG, from the southern agroecological zone, and GR, sourced from the Gera hotspot area, which demonstrated a higher production of conidia and inflicted more severe infections on coffee green berries and seedlings compared to other isolates. This finding aligns with the research conducted by Derso and Waller [5], which reported high conidial production and identified the most aggressive isolate originating from the Rift Valley area around Fishagenet, near Yirgacheffe in southern Ethiopia, as well as the Gera isolate from southwestern Ethiopia. The variability in fungal pathogenicity and the strong correlation between sporulation and pathogenicity offer valuable insights for screening germplasm collections for resistance to this pathogen. Such information is critical for informing breeding programs aimed at developing durable resistance through the use of highly sporulated and virulent isolates. Consequently, the virulent isolate FG, identified in this study from the Fishagenet Rift Valley area of Yirgacheffe district, should be utilized as a benchmark isolate in evaluations of coffee resistance to CBD. Furthermore, the conidial sizes among the *C. kahawae* isolates exhibited considerable variability, both within individual isolates and across the group, indicating that conidial size does not significantly contribute to pathogenicity. This finding is consistent with observations made by Zenebe et al. [12] and Amare et al. [11], who also concluded that conidial size has no bearing on the pathogenic potential of *C. kahawae* isolates in Ethiopia. The high degree of variation in conidial size among isolates highlighted the lack of a significant relationship between pathogenicity and conidial size [25].

## 5. Conclusions

Ethiopia’s coffee production is hindered by various challenges, with diseases being the most significant threat to crop yields. Among these, CBD stands out as the primary concern in the country’s coffee‐growing regions, especially in the south. This study is aimed at characterizing and evaluating the pathogenicity of *Colletotrichum* isolates collected from infected green coffee berries in southern Ethiopia. The findings indicated pathogenic variability among the *Colletotrichum* isolates in their cultural, morphological, incubation periods, and pathogenic characteristics. The Gera (GR) and FG isolates exhibited the most rapid radial growth rates at 4.14 and 4.11 mm/day, respectively, while the MF02 isolate displayed the slowest growth rate at 2.05 mm/day. Notably, isolates FG and GR produced significant amounts of conidia, with counts of 418.12 × 10^4^ and 412.19 × 10^4^ conidia/mL, while the MF02 isolate yielded the lowest amount at 157.21 × 10^4^ conidia/mL. High conidial production is indicative of virulent *C. kahawae* isolates that provide ample inoculum for disease proliferation. Among detected isolates, 13 were found to be pathogenic (*C. kahawae*) to the susceptible coffee variety 370, while the remaining five isolates were found to be nonpathogenic. Moreover, substantial variation in aggressiveness levels was observed among the isolates. Isolates FG from the Yirgacheffe district and GR from Gera recorded the highest (100.00%) disease reaction rate, while five isolates showed the no (0.00%) disease reaction rate. These results underscore the importance of employing aggressive isolates from specific agro ecological zones in the development of resistant coffee varieties, which is crucial for the successful screening of coffee germplasms.

## Conflicts of Interest

The author declares no conflicts of interest.

## Funding

No funding was received for this manuscript.

## Data Availability

Most of the data supporting the findings of this study are included in the article. Additional data are available from the corresponding author upon request.
